# Integrative analysis reveals multiple modes of LXR transcriptional regulation in liver

**DOI:** 10.1073/pnas.2122683119

**Published:** 2022-02-10

**Authors:** Lara Bideyan, Wenxin Fan, Karolina Elżbieta Kaczor-Urbanowicz, Christina Priest, David Casero, Peter Tontonoz

**Affiliations:** ^a^Department of Pathology and Laboratory Medicine, University of California, Los Angeles, CA 90095;; ^b^Department of Biological Chemistry, University of California, Los Angeles, CA 90095;; ^c^Molecular Biology Institute, University of California, Los Angeles, CA 90095;; ^d^Division of Oral Biology and Medicine, School of Dentistry, University of California, Los Angeles, CA 90095;; ^e^Institute for Quantitative and Computational Biosciences, University of California, Los Angeles, CA 90095;; ^f^F. Widjaja Foundation Inflammatory Bowel and Immunobiology Research Institute, Cedars-Sinai Medical Center, Los Angeles, CA 90048

**Keywords:** LXR, nuclear receptor, transcription

## Abstract

The nuclear receptors liver X receptor (LXR) α and β play crucial roles in hepatic metabolism. Many genes induced in response to pharmacologic LXR agonism have been defined; however, the transcriptional consequences of loss of LXR binding to its genomic targets are less well characterized. Here, we addressed how deletion of both LXRα and LXRβ from mouse liver (LXR double knockout [DKO]) affects the transcriptional regulatory landscape by integrating changes in LXR binding, chromatin accessibility, and gene expression. Many genes involved in fatty acid metabolism showed reduced expression and chromatin accessibility at their intergenic and intronic regions in LXRDKO livers. Genes that were up-regulated with LXR deletion had increased chromatin accessibility at their promoter regions and were enriched for functions not linked to lipid metabolism. Loss of LXR binding in liver reduced the activity of a broad set of hepatic transcription factors, inferred through changes in motif accessibility. By contrast, accessibility at promoter nuclear factor Y (NF-Y) motifs was increased in the absence of LXR. Unexpectedly, we also defined a small set of LXR targets for direct ligand-dependent repression. These genes have LXR-binding sites but showed increased expression in LXRDKO liver and reduced expression in response to the LXR agonist. In summary, the binding of LXRs to the hepatic genome has broad effects on the transcriptional landscape that extend beyond its canonical function as an activator of lipid metabolic genes.

## Significance

LXRs are critical regulators of hepatic metabolism and function, but their mechanisms of action at the genome level are incompletely understood. We performed integrated analysis of genome-wide chromatin accessibility, gene expression, and transcription factor binding. We reveal distinct mechanisms of LXR transcriptional regulation of both metabolic and nonmetabolic genes in liver. We show that LXR can both activate and repress genes and that LXR binding impacts the activity of other transcription factors.

Liver X receptor (LXR) α and LXRβ (encoded by *Nr1h3* and *Nr1h2*) play important roles in hepatic lipid metabolism. LXRs are crucial for the lipogenic response to feeding as regulators of *Srebf1*, *Fasn*, and *Scd1* (1–3). LXRs play a role in phospholipid remodeling via control of *Lpcat3* expression (4, 5). In liver as in other tissues, LXRs are also central to cholesterol homeostasis. Activated LXRs induce genes involved in cholesterol efflux, such as those encoding ABCA1, ABCG5, and ABCG8, block low-density lipoprotein (LDL) uptake through IDOL, and promote cholesterol conversion to bile acids through CYP7A1 (6–9). Beyond metabolism, LXRs have been shown to regulate immune responses in macrophages, including those in the liver (10–12). LXRα is a lineage-determining factor for Kupffer cells and necessary to maintain gene expression defining their identity (13–15).

LXRs are activated by oxysterols such as 27-hydroxycholesterol and 4β-hydroxycholesterol and intermediates in the cholesterol biosynthetic pathway, such as desmosterol (16–19). Loss of LXRs leads to pathological cholesterol accumulation in liver when mice are fed a high-cholesterol diet (20). In the absence of excess dietary cholesterol, the primary consequences of LXR deletion in liver are perturbations in fatty acid and phospholipid metabolism (21, 22). Many studies have used synthetic agonists such as GW3965 and T0901317 as tools to investigate the role of LXRs in hepatic gene expression (23). Activation of LXRs with synthetic agonist improves atherosclerosis and glucose tolerance, but also increases hepatic lipogenesis (24–26). Chromatin immunoprecipitation with sequencing (ChIP-Seq) studies have defined LXR-binding sites in the hepatic genome and noted increased LXR binding to lower-affinity DNA sites in the presence of a synthetic agonist (27).

Given the widespread use of synthetic agonists to identify LXR-responsive genes, it is not surprising that LXRs have been characterized primarily for their roles as ligand-dependent activators. Recent studies using alternative approaches and genome-wide techniques have revealed multiple modes of LXR gene regulation. Ramón-Vázquez et al. defined three modes of LXR action in macrophages. The first is the classical mode of agonist-activated genes; the second is a derepression mode, in which target genes are up-regulated both in response to agonist and in the absence of LXRs; and the third is a pharmacologically nonresponsive mode for genes that require LXRs for expression but do not change in response to agonist (28). Systematic analyses of different modes of LXR action on gene expression in vivo in key metabolic tissues have not yet been performed.

LXRs bind to DNA as obligate heterodimers with retinoid X receptor (RXR). The canonical LXR-binding site (LXRE) is a repeated nuclear receptor half-site motif (AGGTCA) separated by four nucleotides (DR4) (29). LXR liver ChIP-Seq studies have suggested broader LXR binding to genomic sites other than DR4 motifs. One notable limitation of genome-wide bioinformatic approaches, however, is the degenerate nature of many LXREs and peroxisome proliferator-activated receptor (PPAR) response elements (PPREs), which makes motif identification challenging. Many biologically critical LXREs and PPREs are not perfect DR4 or DR1 elements (30, 31). Studies integrating genomic analyses of multiple nuclear receptors, including LXR and PPARα, have shown greater overlap between receptor targets than expected (27). Such extensive cobinding has been proposed to lead to functional cross-talk. In support of this idea, loss of LXRs was shown to diminish activation of PPARα targets in response to PPARα agonist (22).

The dynamics of the transcriptional landscape in response to LXR binding to the genome are largely unknown. Tools such as assay for transposase accessible chromatin (ATAC-Seq) can provide a bridge between transcription factor (TF) binding and gene expression by revealing dynamic changes in chromatin accessibility (32). In this study, we investigated the transcriptional dynamics of LXR in mouse liver. We performed RNA sequencing (RNA-Seq) and ATAC-Seq on livers of LXRα and LXRβ double-knockout (LXRDKO) mice to characterize the effect of loss of LXRs on the transcriptional landscape. Incorporating available LXR liver ChIP-Seq data, we identified how LXR-binding sites changed and how those changes related to differential gene expression. We also profiled the differences in activity of other transcription factors in response to loss of LXRs using the accessibility of their binding motifs. We integrated our results from the LXRDKO model with data from synthetic agonist treatment studies (33) to define distinct modes of LXR action in the liver, including the ability to act as a ligand-dependent repressor. These findings contribute to a more thorough understanding of how LXRs impact the transcriptional landscape and orchestrate hepatic metabolism.

## Results

### Transcriptional Changes in Liver of LXRDKO Mice.

We performed RNA-Seq on livers of whole-body LXRα/β LXRDKO mice to profile transcriptional changes provoked by the absence of these transcription factors. We identified 246 up-regulated and 321 down-regulated genes using an adjusted *P* value of <0.05 (*SI Appendix*, Fig. S1*A*). Many classical LXR targets were down-regulated, including *Srebf1* and *Fasn* (*SI Appendix*, Fig. S1 *B* and *C*) (1, 3, 34). Down-regulated genes associated with lipid metabolism pathways as expected and overlapped substantially with the set of direct LXR target genes annotated in publicly available ChIP-Seq datasets (*SI Appendix*, Fig. S2 *A* and *B*). In addition, lipid metabolism genes associated with PPARα were down-regulated in LXRDKO livers. Macrophage and Kupffer cell marker genes, such as *Cd5l*, *Cd163*, and *Clec4f* were also among the most down-regulated genes. Reduced expression of these genes likely reflects a change in the immune cell profile in LXRDKO liver, as LXR is known to be important for Kupffer cell identity (10, 14, 15). Interestingly, most of the genes up-regulated in the absence of LXR expression were not established LXR targets and did not have obvious links to lipid metabolism (*SI Appendix*, Fig. S1 *B* and *C*). These genes were enriched for pathways including cysteine and methionine metabolism and DNA repair/p53 response (*SI Appendix*, Fig. S2*A*).

### Changes in Chromatin Accessibility in Liver of LXRDKO Mice.

**Fig. 1.**
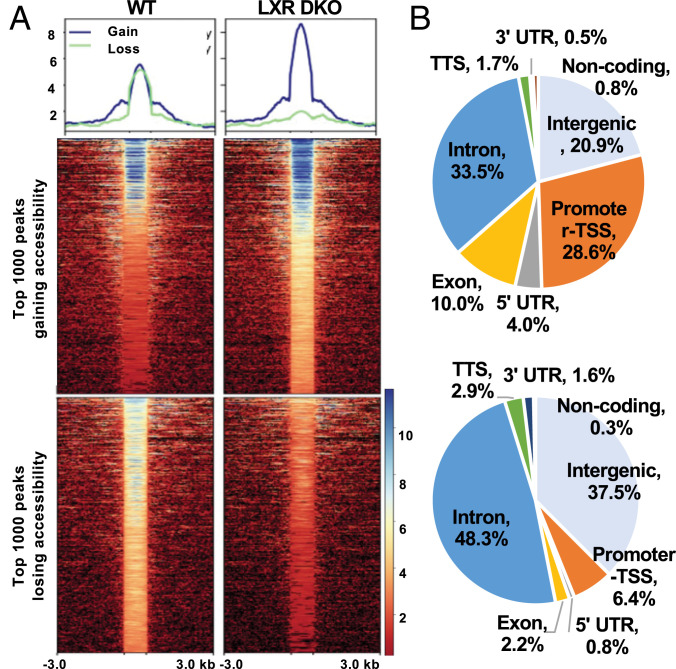
Global chromatin accessibility changes in LXRDKO liver. (*A*, *Top*) Average normalized ATAC-Seq signal intensity for top 1,000-ranked peaks changing in accessibility in WT and LXRDKO samples. (*Bottom*) Heatmap of signal distribution around ATAC-Seq peak summits, for the same peaks. (*B*) Pie charts showing distribution of genomic features among the top 1,000 peaks with largest loss and gain in LXRDKO liver.

We next aimed to further delineate how the absence of LXRs induced the observed changes in gene expression. To understand how changes in transcription in the absence of LXRs related to genome-wide chromatin accessibility, we performed ATAC-Seq to quantify genome-wide chromatin accessibility on the livers of the same mice used in the transcriptomics analysis above. It has been reported that changes in accessibility in response to perturbation in the liver are less dramatic than in other tissues (15, 35, 36). A total of 95,342 peaks were detected across the samples. Correlation between samples is shown in *SI Appendix*, Fig. S3 *A* and *B*. We ranked our ATAC-Seq peaks based on the absolute change in accessibility between LXRDKO and wild-type (WT) livers. After filtering out peaks with very weak signals, 73,597 peaks remained, of which 57.60% had passed an irreproducible discovery rate of 1e-6 (37). We viewed the top 1,000 peaks with increased or decreased accessibility in LXRDKO livers to detect overall patterns in the changes in chromatin accessibility. Genomic sites that lost the most accessibility in the LXRDKO liver largely became inaccessible in LXR liver (Fig. 1*A*). In comparison, sites that gained the most accessibility in the LXRDKO liver were already open in WT samples and became even more accessible in LXRDKO livers.

Top peaks that lost accessibility were enriched in intergenic and intronic regions (Fig. 1*B*). In comparison, top peaks that gained accessibility in the absence of LXRs were more likely to be found in promoter and exonic regions of the genome than those that lost accessibility. Consistent with this observation, top peaks gaining accessibility in the absence of LXRs were more likely to be located within 1 kb of transcription start sites (TSSs), while top peaks losing accessibility were enriched in regions >10 kb away from TSSs (*SI Appendix*, Fig. S3*C*). These findings broadly suggest reductions in potential enhancer activity and increases in direct promoter activity on a range of genes in LXRDKO livers.

### Integrating Gene Expression and Chromatin Accessibility.

Average accessibility across the gene was decreased in the genes down-regulated in the absence of LXR in comparison to up-regulated ones (*SI Appendix*, Fig. S3*D*). Genes down-regulated in the absence of LXR were more likely on average to lose accessibility in their intergenic and intronic peaks, compared to those whose expression increased or did not change (*SI Appendix*, Fig. S3*E*). On the other hand, promoter peaks in genes up-regulated in LXRDKO liver were more likely to gain accessibility, compared to those whose expression decreased or did not change. These results agree with the enrichment of intergenic and intronic regions in the top peaks losing accessibility and the enrichment of promoters for top peaks gaining accessibility. Pathway enrichment analysis revealed that the set of genes proximal to top peaks losing accessibility in LXRDKO liver were enriched in lipid metabolism pathways, in agreement with the types of genes down-regulated (*SI Appendix*, Fig. S4). Genes proximal to top peaks gaining accessibility were enriched for pathways other than lipid metabolism (e.g., endocytosis). These observations support a degree of correlation between chromatin accessibility and gene expression in LXRDKO liver.

### Correlation of Accessibility, LXR Binding, and Gene Expression.

**Fig. 2.**
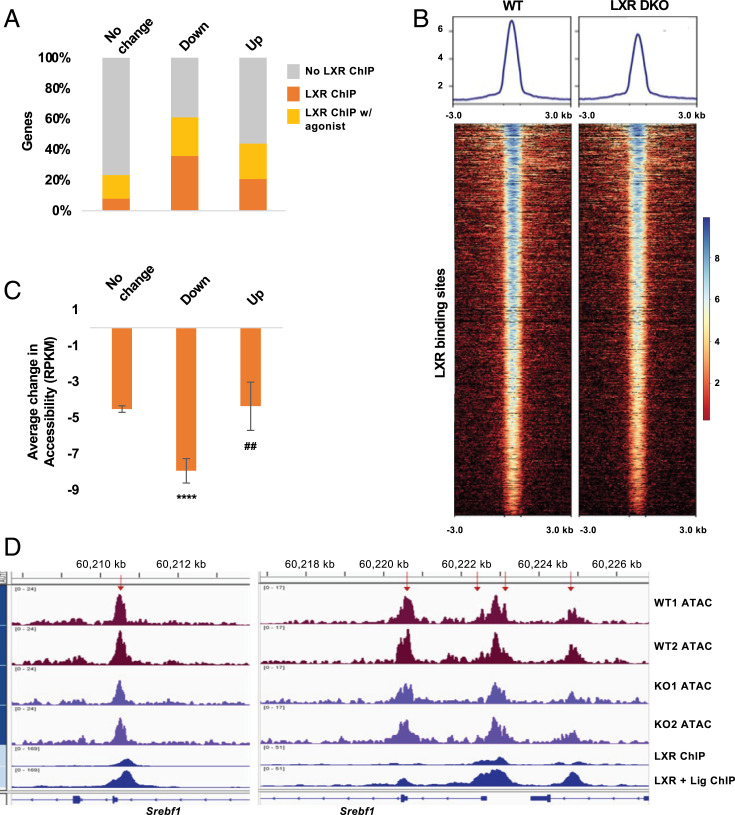
Correlation of LXR binding, gene expression, and chromatin accessibility. (*A*) Comparison of the proportion of genes with LXR liver ChIP-Seq and LXR liver + T0901317 ChIP-Seq binding (27) among genes whose expression is down-regulated, up-regulated, and unchanged in LXRDKO liver. (*B*) ATAC-Seq signal intensity across LXR (basal)-binding sites in WT and LXRDKO samples. Average signal profile is plotted on *Top*. (*C*) Average change in LXR-binding site accessibility for peaks proximal to genes whose expression is down-regulated, up-regulated or unchanged in LXRDKO liver in comparison to WT. ****, indicates the comparison to genes with unchanged expression. ##, indicates the comparison between downregulated and upregulated genes. (*D*) Example of ATAC-Seq signal on LXR-binding sites in the alternative first exon (*Left*) and promoter (*Right*) of the *Srebf1* locus. Peaks that on average lost accessibility for at least 10 RPKM in LXRDKO compared to WT are indicated with red arrows. Publicly available LXR liver ChIP-Seq data are aligned alongside the ATAC-Seq tracks (27).

To further examine changes in accessibility occurring at LXR-binding sites, we integrated our RNA-Seq and ATAC-Seq results with LXR ChIP-Seq data from livers of mice treated with vehicle (basal) or the LXR agonist T0901317 (27). Our analysis revealed that 35.8% of the down-regulated genes and 20.7% of the up-regulated genes in LXRDKO livers were putative LXR ChIP-Seq targets (compared to 7.8% of the nondifferentially expressed genes; Fig. 2*A*). When we included genomic LXR-binding sites observed only with T0901317 treatment (27), more than half of the down-regulated genes (61.1%) and 43.9% of the up-regulated genes had LXR-binding sites. In short, the majority of the differentially expressed genes in LXRDKO livers had LXR binding detected by ChIP-Seq. However, only a small fraction of the genes associated with LXR liver ChIP-Seq peaks was differentially expressed in LXRDKO livers (7.1% of the vehicle treated and 4.6% of the T0901317-treated ChIP-Seq sites, *SI Appendix*, Fig. S5*A*). Among genes with LXR-binding sites, genes that were differentially expressed between WT and LXRDKO mice tended to have a higher number of LXR-binding sites compared to genes whose expression did not change in LXRDKO liver (*SI Appendix*, Fig. S5*B*). This suggests that only a small subset of LXR-binding sites in liver is functionally required for hepatic gene expression.

The overall accessibility across LXR-binding sites was reduced in LXRDKO livers; 71.57% of LXR-binding sites lost some accessibility (Fig. 2*B* and *SI Appendix*, Fig. S5*C*). A majority of the ATAC peaks at LXR-binding sites located at intergenic and intronic regions in LXRDKO liver showed a decrease in accessibility, but that trend was not observed for peaks located at promoter regions (*SI Appendix*, Fig. S5*D*). Thus, the degree to which LXR-binding sites changed in accessibility in LXRDKO liver was influenced by their locations in relation to individual genes.

Integrating the expression, binding, and chromatin accessibility data, LXR-binding sites associated with down-regulated genes were less accessible in LXRDKO liver than those associated with genes whose expression did not change or were up-regulated (Fig. 2*C*). For instance, one context where LXR binding is known to be functionally important is at the *Srebf1* locus (34). The regulatory regions of *Srebf1* contain multiple LXR-binding sites (Fig. 2*D*), including one at the alternative promoter for *Srebf1c* (*Left*). All of the LXR-binding sites at this gene lost some accessibility in the LXRDKO samples compared to controls, accompanying the down-regulation of the gene.

### Loss of LXR Affects Accessibility at Binding Sites for Other Transcription Factors.

**Fig. 3.**
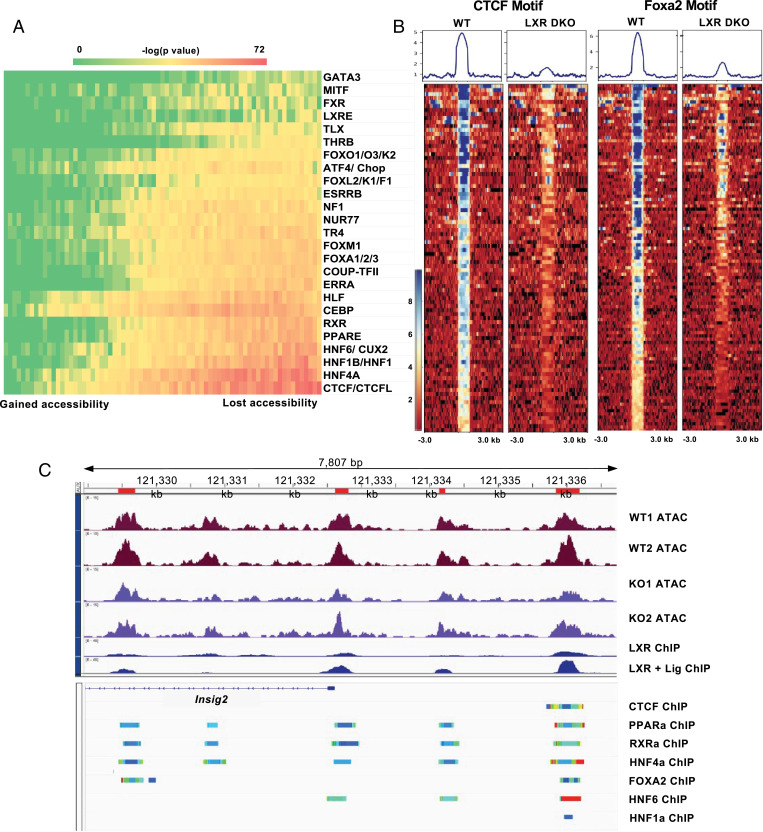
Loss of LXR affects chromatin accessibility at other transcription factor–binding sites. (*A*) Heatmap of motif accessibility across all ATAC-Seq peaks ranked and binned based on the accessibility difference between LXRDKO and WT samples. Shown are 73 bins each containing ∼1,000 peaks. The heatmaps represent the enrichment *P* value obtained from known motif analysis. Transcription factors are grouped based on motif similarity (>90%). Only motifs that were enriched in peaks that lost accessibility in LXRDKO liver are shown. (*B*) ATAC-Seq signal intensity heatmap and profiles across peaks associated with CTCF (*Left*), and FoxA2 (*Right*) motifs, among the top 1,000 peaks losing accessibility in LXRDKO livers. (*C*) Browser view of *Insig2* locus showing WT and LXRDKO ATAC-Seq normalized signal alongside LXR ChIP-Seq data (27). *Below* the reference gene are ChIP-Atlas tracks presenting aggregate liver ChIP-Seq data for selected transcription factors (74).

Changes in chromatin accessibility at transcription factor–binding sites may reflect a change in transcription factor activity. To analyze these trends across the genome, we ranked all of our ATAC-Seq peaks based on changes in accessibility between LXRDKO and WT samples and binned them into equal-sized bins (∼1,000 peaks each). We performed motif enrichment analysis for known binding motifs (see *Methods* for details) for all of the bins. We then displayed the enrichment of each of the transcription factor motifs across all bins in a heatmap (Fig. 3*A*). This method allowed us to visualize the difference in enrichment of each motif both across bins and compared to other motifs. The results showed a gradual increase in enrichment of binding motifs in relation to changes in chromatin accessibility in LXRDKO liver. We identified several transcription factors whose binding motif became less accessible in LXRDKO liver. Motifs predicted to bind CTCF/CTCFL, the nuclear receptor family, HNF1/HNF1B, HNF6/CUX2, the FOX family, and the ATF4/CHOP family showed the strongest enrichment in peaks that lost accessibility in LXRDKO liver. Among nuclear receptor motifs, the DR1 motif (bound by PPARα, HNF4α, and RXR) was the most strongly enriched, but the DR4 motif (bound by LXR and thyroid hormone receptor [TR]) and the nuclear receptor half-site motif (recognized by ERRs, Coup-TFII, and others) were also enriched. Many of these transcription factor motifs are primarily present in intergenic and intronic regions. Even among intergenic and intronic peaks, these motifs were enriched in peaks that were specifically losing accessibility in LXRDKO liver (*SI Appendix*, Fig. S6*A*).

We further examined the transcription factor motifs associated with the top 1,000 ATAC peaks that lost accessibility in LXRDKO liver (Fig. 3*B*). A number of peaks associated with CTCF motifs lost almost all signal in LXRDKO liver, indicating largely inaccessible CTCF-binding sites. In comparison, peaks associated with PPAR motifs and FOXA2 motifs showed strong reductions in ATAC signal intensity but still retained some accessibility (Fig. 3*B* and *SI Appendix*, Fig. S6*B*). This result implies that loss of CTCF binding may lead to the closing of these peaks. To ensure that the motifs we identified were independently changing, we examined the peaks among the top bins that lost accessibility in LXRDKO liver with these motifs. Each family motif was present on a unique set of peaks with some overlap with other transcription factor families (*SI Appendix*, Fig. S6 *C*, *Left*). This suggests that there is specificity to the reduction of motif accessibility for each of these transcription factor families, and that the reduction of accessibility of one transcription family was not completely dependent on another transcription factor family. With the exception of a modest decrease in PPARα and modest increase in Foxa2 expression in the LXR DKO samples, the expression of most of these transcription factors themselves was not different between groups, suggesting that the changes in their motif accessibility were not likely to be due to differences in transcription factor abundance (*SI Appendix*, Fig. S6*D*).

We further assessed changes in accessibility of some of the motifs via footprinting (38). This approach measures transcription factor–binding activity by quantifying the protection of the binding site from sequencing. The accessibility of predicted binding sites for HNF1B and HNF6A were reduced across the LXRDKO liver genome compared to control (*SI Appendix*, Fig. S7 *A* and *B*). Although this method was not as sensitive, it nevertheless provided independent validation of some of the observations shown in Fig. 3*A*.

To address how the loss of LXR affected the activity of other transcription factors specifically at its target genes, we performed a similar analysis on the top bins of ATAC peaks proximal to a putative LXR-binding gene that lost accessibility in LXRDKO liver. When we clustered genes associated with each transcription factor motif, we observed patterns of motif cooccurrence across different families (*SI Appendix,* Fig. S6 *C*, *Right*). This suggests that a number of transcription factors were collectively losing accessibility in LXR target genes. As an example, the *Insig2* locus has a number of LXR-binding sites that became less accessible in LXRDKO liver. Based on available liver ChIP-Seq datasets, each of the peaks associated with LXR binding was also predicted to bind to combinations of other transcription factors, including CTCF, PPAR, RXR, HNF4A, FOXA2, HNF6, and HNF1 (Fig. 3*C*), exemplifying how the loss of LXR could impact the potential binding of other transcription factors to the same gene. Our analysis revealed instances of independent and collective loss of activity of these transcription factors on LXR-binding genes.

### Nuclear Factor Y (NF-Y) Motifs Are More Accessible in the Absence of LXRs.

**Fig. 4.**
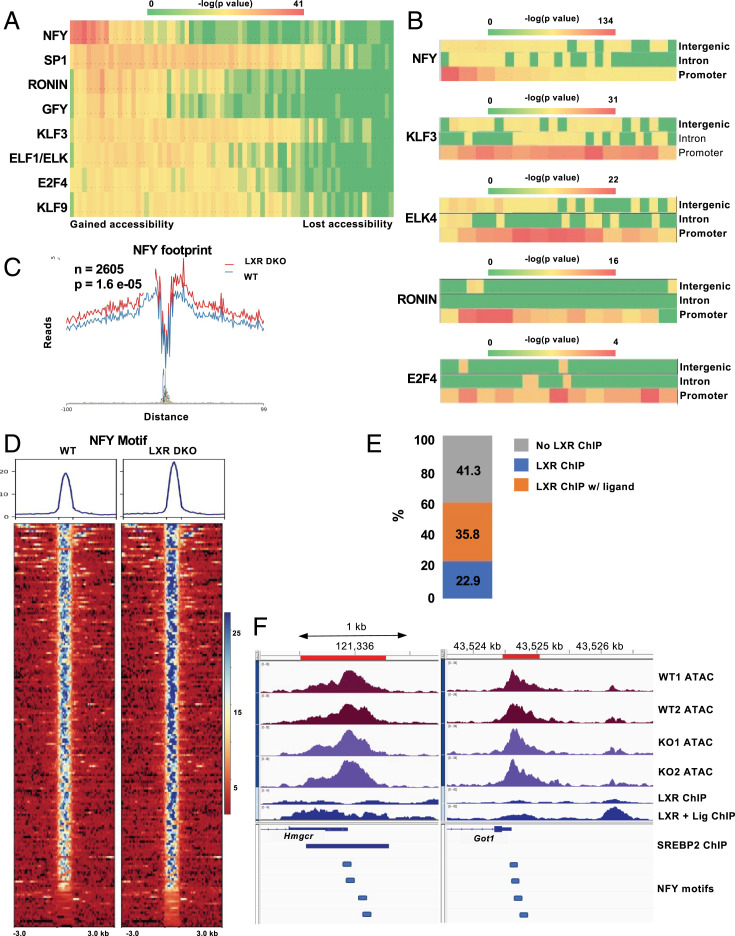
Increased accessibility of NF-Y motifs in LXR-deficient liver. (*A*) Heatmap of motif accessibility across all ATAC-Seq peaks ranked and binned based on differences between LXRDKO and WT. Shown are 73 bins each containing ∼1,000 peaks. The heatmap represents the enrichment *P* value obtained from known motif analysis. Transcription factors are grouped based on motif similarity (>90%). Only motifs that were enriched in peaks that gained accessibility in LXRDKO liver are shown. (*B*) Heatmaps of motif enrichment of selected overrepresented transcription factors across binned intergenic, intronic, and promoter ATAC-Seq peaks based on change in accessibility. (*C*) Footprint of the NF-YA motif in WT and LXRDKO ATAC-Seq samples using HINT-ATAC. (*D*) ATAC-Seq signal intensity heatmap and profile of peaks with NF-Y motif among the top 1,000 peaks with the largest gains of accessibility. (*E*) Within the top 1,000 peaks that gained accessibility in LXRDKO, proportion of genes with increased NF-Y motif accessibility that also have LXR binding. (*F*) Browser view of peaks with increased NF-Y motif accessibility, including the promoter regions of *Hmcgr* (*Left*) and *Got1* (*Right*). Publicly available SREBP2 liver ChIP-Seq and NF-Y motif locations are aligned below the gene annotation (77).

Fewer transcription factor motifs were enriched in ATAC peaks that gained accessibility in LXRDKO liver (Fig. 4*A*). Interestingly, many of these binding sites share a core ETS motif and are known to appear frequently in promoter regions. Peaks in promoter regions were overrepresented among those that gained accessibility in the absence of LXRs (Fig. 1*B*). Among the promoter peaks, the NF-Y motif was particularly prevalent among those that gained accessibility in LXRDKO liver (Fig. 4 *A* and *B*). By contrast, ETS family motifs were enriched across promoter regions without a preference for sites that gained accessibility upon loss of LXR. Footprinting analysis validated this finding (Fig. 4*C*). The NF-Y footprint was more accessible across the LXRDKO liver ATAC-Seq sample compared to control. Peaks at which NF-Y motifs gained accessibility were already accessible in WT samples, but became even more accessible in LXRDKO samples (Fig. 4*D*).

A majority of genes with increased NF-Y accessibility had an LXR-binding site (either basal or with agonist treatment) (Fig. 4*E*). Moreover, the NF-Y motif was enriched among the LXR-binding promoter peaks that were increased in accessibility. This could indicate that the absence of LXR could be leading to compensatory increased NF-Y binding at these LXR target genes. Genes proximal to NF-Y motifs that gained accessibility in LXRDKO liver were enriched for pathways related to cell cycle, NF-κB signaling, and cholesterol synthesis (*SI Appendix*, Fig. S7*C*), and included SREBP2 targets such as *Hmgcr*, *Hmgcs1*, *Sqle*, and *Fdps*. For instance, the *Hmgcr* promoter was on average more accessible in LXRDKO liver (Fig. 4*F*). The peak in this region overlaps with an SREBP2-binding site and contains four NF-Y–binding motifs. Interestingly, among genes with increased NF-Y accessibility, only a small proportion was differentially expressed between WT and LXR DKO liver (3.36%, accounting for 15.8% of all up-regulated genes in LXR DKO) (*SI Appendix*, Fig. S7*D*). As an example, *Got1* was up-regulated and its promoter (with four NF-Y motifs) was on average more accessible in LXRDKO liver (Fig. 4*F*).

**Fig. 5.**
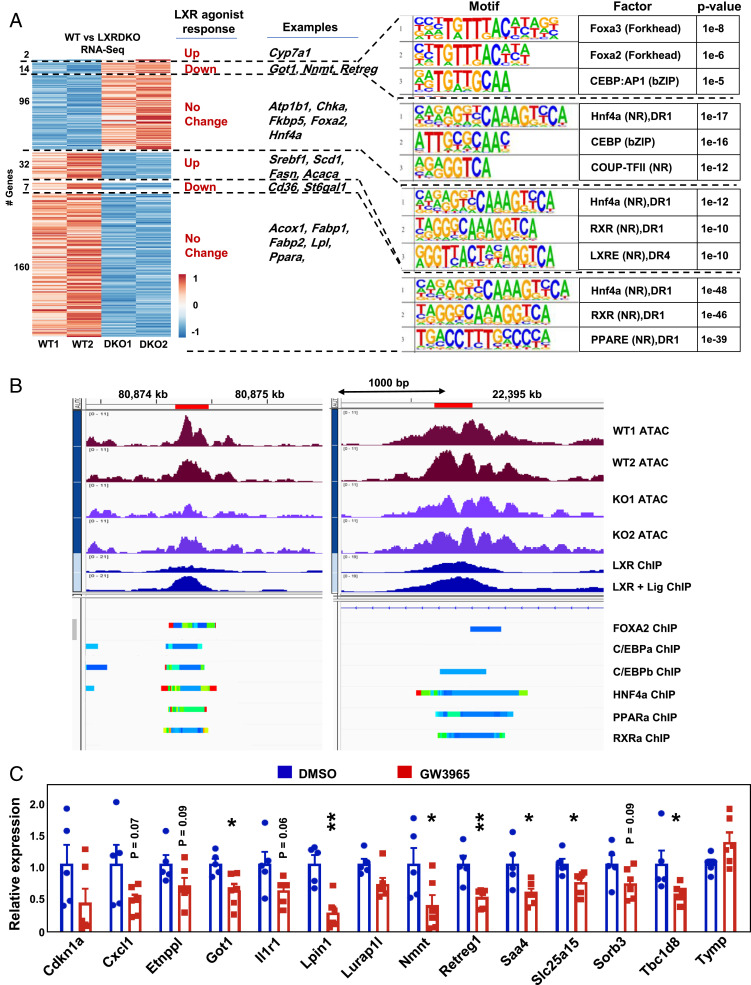
LXR can act as a ligand-dependent and -independent repressor. (*A*) Heatmap of normalized counts of differentially expressed genes with an LXR ChIP-Seq–binding site. Unit variance scaling was used for scaling rows. Genes are arranged according to their behavior in response to agonist treatment (combining publicly available GW3965 and T0901317 treatment results). Highlighted are selected genes in each segment. The top three results from known motif analysis for each segment are shown in the following order: 1) Up-regulated in LXRDKO samples and down-regulated by agonist, 2) up-regulated in LXRDKO and not changed by agonist, 3) down-regulated in LXRDKO and up-regulated by agonist, 4) down-regulated in LXRDKO and not changed by agonist. (*B*) Example regions in which peaks with LXR-binding motifs were on average less accessible in LXRDKO liver, for genes where LXR was acting as a repressor. Intergenic region associated with the Lurap1l gene (*Left*) and intronic region associated with Slc25a15 (*Right*). (*C*) Independent validation of 14 ligand-repressed genes by qPCR assessment from livers of acute GW3965-treated mice (*n* = 5 to 6).

### Distinct Modes of LXR Transcriptional Regulation in Liver.

Many studies on LXRs have focused on their functions as ligand-activated transcription factors, using pharmacological tools such as the potent synthetic agonists GW3965 and T0901317 (26, 39, 40). Our analysis of global accessibility changes induced by loss of LXR supported this major mode of LXR action, but also revealed additional mechanisms. We integrated datasets for genes differentially expressed in liver in response to GW3965 or T0901317 treatment with LXR ChIP-Seq data and our RNA-Seq data (33). We found that the expression of a majority of the differentially expressed genes in WT vs. LXRDKO liver was not altered by agonist treatment (Fig. 5*A*). This was true even for those genes predicted to have LXR binding by ChIP-Seq. This observation suggests distinct basal and pharmacological ligand-dependent functions for LXRs at individual genes.

We next focused on genes that were regulated both by synthetic agonist and the presence or absence of LXRα/β. A majority of these genes was regulated in opposite directions by agonist treatment and LXR deletion (Fig. 5*A*). We identified 32 genes that were down-regulated in LXRDKO liver and induced by agonist treatment in WT liver (Fig. 5*A*). This set was enriched for classical LXR targets mostly involved in fatty acid metabolism, including *Srebf1*, *Scd1*, *Acaca*, *Fasn*, and *Lpcat3*. ATAC peaks at these LXR-binding sites were enriched for DR1 and DR4 nuclear receptor motifs. Additionally, LXR-binding sites for these pharmacological ligand-activated genes were more likely to be located in promoter regions in comparison to pharmacological ligand-unresponsive genes down-regulated in LXRDKO liver (*SI Appendix*, Fig. S8*A*).

By contrast, a substantial subset of genes down-regulated in LXRDKO liver with putative LXR binding but no transcriptional response to agonist were enriched for canonical PPARα targets such as *Acox1*, *Acsl5*, and *Fabp1* (Fig. 5*A* and *SI Appendix*, Fig. S8*B*). The LXR-binding sites for these genes were enriched for the nuclear receptor DR1 motif (Fig. 5*A*). This observation suggests that LXR may associate widely with DR1 nuclear receptor motif sites and thereby contribute to the expression of canonical PPARα genes that do not respond to pharmacologic LXR agonist.

The LXRE/DR4 motif was enriched among the set of intergenic and intronic ATAC peaks with putative LXR-binding sites that showed the largest loss of accessibility in LXRDKO liver (*SI Appendix*, Fig. S8*C*). The DR1 motif was enriched across all intergenic and intronic LXR-binding sites regardless of their change in accessibility in LXRDKO livers. Thus, the DR4/LXRE motif is strongly associated with classical LXR targets that are up-regulated by agonist treatment and down-regulated in the LXRα/β knockout and with LXR-binding sites that lose accessibility with the loss of LXR. The data further suggest that, outside of these canonical LXR targets, LXR can bind to nuclear receptor motifs more broadly, including at DR1 motifs.

We also identified 14 genes that were up-regulated in the absence of LXRs, down-regulated with agonist treatment, and had putative LXR-binding sites by ChIP-Seq (Fig. 5*A*). These genes represent potential targets for direct ligand-dependent repression by LXRs. This mode of regulation is known to occur with certain other nuclear receptors, such as TR, but has not been rigorously documented for LXRs. LXR-binding sites for these ligand-repressed genes were more likely to be located in promoter regions (*SI Appendix*, Fig. S8*A*). These LXR-repressed genes were involved in various cellular functions not focused on lipid metabolism. Interestingly, ATAC peaks associated with the LXR-binding sites in these repressed genes were enriched for the FOXA and C/EBP motifs. Overall accessibility of these LXR-binding sites were decreased in LXRDKO liver (*SI Appendix*, Fig. S8*D*). As an example, *Slc25a15* and *Lurap1l* had increased expression in LXRDKO liver and decreased expression with LXR agonist treatment. Their loci have LXR-binding sites that were on average reduced in accessibility in LXRDKO livers and have putative binding sites for FOXA2, C/EBP, and other nuclear receptors such as HNF4a and PPARα detected by ChIP-Seq (Fig. 5*B*). By comparison, genes up-regulated in LXRDKO liver that lack the transcriptional response to the agonist included genes involved in cysteine and methionine metabolism and genes encoding for transcription factors such as *Foxa2* and *Hnf4a* (Fig. 5*A*). These finding suggests that FOXA1/2 binding could be important for LXR ligand–dependent repressor function. In accordance with our findings, published data suggest that loss of hepatic *Foxa2* abolishes the down-regulation of some of these genes (*Cxcl*, *Etnppl*, *Got1*, *Nnmt*, *Slc25a15*, *Tbc1d8*, and *Tymp*) by LXR agonist (GSE149075) (33).

To further validate the ability of LXR agonists to repress gene expression through direct LXR binding, we treated WT mice with GW3965 and measured gene expression by qPCR (Fig. 5*C*). Of the 14 genes tested, 8 were reduced by GW3965 treatment, and 4 trended down (*P* value <0.1). Independent confirmation of the down-regulation of these predicted targets supports the conclusion that LXRs are capable of acting as ligand-dependent repressors.

## Discussion

In this study, we assessed the implications of loss of LXR expression in mouse liver for gene expression, chromatin accessibility, and transcription factor activity. Unlike fork head factors, LXRs are not known to be pioneer factors that play key roles in establishing regions of open chromatin. Accordingly, the changes in chromatin accessibility we observed with loss of these nuclear receptors, especially on LXR-binding sites, were rarely dramatic; i.e., complete closing of an existing peak or opening of a new peak. A majority of the genes differentially expressed between WT and LXRDKO liver had LXR-binding sites, suggesting the change in their expression was likely to be a direct consequence of loss of LXR binding. At the same time, a majority of the genes differentially expressed between WT and LXRDKO liver did not change in WT mice treated with synthetic LXR agonist. This finding suggests that many LXR-binding sites do not transduce ligand-dependent signals or are active with basal levels of endogenous ligands. Such LXR-binding sites appear necessary to maintain expression of their target genes but do not respond to pharmacological activation, perhaps due to specific coactivator requirements. A similar disconnect between basal nuclear receptor activity and synthetic ligand response has been previously observed in macrophages (28). It would be interesting to determine whether challenging mice with a high-cholesterol diet, which would provide a higher concentration of endogenous sterol ligands, would alter the pattern of gene responses.

An important limitation of our study is the use of whole liver tissue. Our RNA expression and chromatin accessibility experiments incorporate signals from hepatocytes and nonparenchymal cells such as Kupffer cells, liver sinusoidal endothelial cells, stellate cells, and other cell types. Changes in gene expression in these nonparenchymal cells and/or shifts in the proportion of these cells present in LXRDKO liver may contribute to the results of the RNA-Seq and ATAC-Seq analyses. In particular, LXRα is known to be crucial for Kupffer cell identity (14, 15), and LXR-deficient liver has been reported to display an altered profile of immune cells, especially in the context of inflammation (41, 42). In agreement with these prior findings, genes highly expressed in Kupffer cells such as *Cd5l* and *Clec4f* showed reduced expression in LXRDKO liver. LXRs have also been reported to affect the capillarization of sinusoidal endothelial cells and the ability of stellate cells to contribute to fibrosis in response to injury (43, 44). Further dissection of the contributions of different cell types to the overall phenotype of LXR-deficient livers will require additional studies, including single-cell RNA-Seq and single-cell ATAC-Seq.

Genes down-regulated in response to LXR deletion in our study were enriched for classical LXR targets related to fatty acid metabolism, including *Srebf1*. Thus, the presence of LXRs on the regulatory regions of these genes appears to be required for their basal expression. Interestingly, however, LXR target genes related to cholesterol metabolism and efflux (such as *Abca1*, *Abcg5*, *Abcg8*, and *ApoA1*) were generally not differentially expressed between WT and LXRDKO liver. Although LXR binding is not required for the basal expression of these genes, prior studies have shown that LXRs mediate induction of these genes in liver in response to synthetic LXR ligand or cholesterol diet challenge (7, 8, 45). This separation is consistent with a primary role for hepatic LXRs in the basal state in fatty acid metabolism and roles in both fatty acid and cholesterol metabolism for LXR in the setting of high ligand concentration (23).

Among genes that responded to both synthetic agonist treatment and LXR deletion in our analysis, most responded in opposite directions. Classical LXR target genes had one or more LXR-binding sites associated with a DR4 or DR1 motif, were reduced in expression with the loss of LXR, and were increased in expression with LXR agonist treatment. Unexpectedly, we also identified a small set of genes that were repressed by LXR in the basal state and in response to synthetic agonist. These genes had putative LXR-binding sites by ChIP-Seq (27), showed increased expression in LXRDKO liver, and showed decreased expression with LXR agonist treatment of WT mice (33). Such a direct ligand-dependent repressor function for LXR has not been demonstrated in liver previously. Analysis of the ATAC peaks associated with LXR binding revealed that the FOXA motif was common to these genes repressed by LXR agonist. Our ATAC-Seq results showed decreased FOXA motif accessibility across the genome in LXRDKO liver, despite increased expression of the *Foxa2* gene. The pharmacological repression by LXR agonist was dependent on the presence of Foxa2 for half of the genes showing direct ligand-dependent repression from our dataset (33). Kain et al. (33) demonstrated the importance of FOXA2 for synthetic ligand-dependent activation of LXR. Our data suggest an additional role for FOXA2 in the ligand-dependent repressor function of LXR.

Our data also provide evidence that the loss of LXRs from the liver affects the activity of other transcription factors. Undoubtedly, alterations in lipid metabolism upon loss of LXRs contributes to some of the gene expression changes observed, such as the reduction in fatty acid synthesis due to loss of *Srebf1* expression (3). Reduced availability of fatty acids would be expected to reduce ligand activation of PPARα. At the same time, we also found evidence of cooperation between LXR and other transcription factors on the regulatory regions of individual genes. One of the most prominent factors whose motif lost accessibility in our LXRDKO dataset was PPARα. Interestingly, the expression of both PPARα target genes and *Ppara* itself was reduced in LXRDKO liver. This finding argues against a competition between PPARα and LXR and indicates that the presence of LXR is necessary for PPARα signaling. Ducheix et al. have noted that the impact of the PPARα agonist fenofibrate on PPARα target genes was decreased in LXRDKO liver (22). Many genes share LXR- and PPARα-binding sites (27), suggesting direct cooperation of LXR and PPARα in their regulation. Many ATAC peaks associated with LXR binding are also associated with binding of other transcription factors such as FOXA2 and HNF6 (Fig. 3*C*). Such regions resemble previously described transcription factor hotspots, which function as superenhancers (46). The reduced accessibility of these sites in LXRDKO liver supports the idea of cooperation between LXRs and other factors thereon.

Other global changes in the LXRDKO liver included increased accessibility of promoter regions and decreased accessibility of intergenic and intronic regions, suggesting a reduction in enhancer activity. This pattern was particularly evident for the intergenic and intronic regions of genes whose expression was down-regulated and for the promoter regions of those up-regulated in LXRDKO liver. The CTCF motif was enriched among the intergenic and intronic regions that lost accessibility in LXRDKO liver. In a recent paper, ATAC-Seq of hearts from CTCF knockout mice showed decreased accessibility in intergenic and intronic regions and increased accessibility in promoter regions (47). A reduction in CTCF activity could thus contribute to the changes in the intergenic and intronic accessibility in the absence of LXRs.

Loss of LXR also appeared to provoke compensatory responses at promoters. In particular, NF-Y motifs broadly increased in accessibility in LXRDKO liver compared to WT. This motif was enriched among promoters already accessible in WT liver that became more accessible in LXRDKO liver. NF-Y is known for its role in maintaining the accessibility of promoter regions and protecting them from nucleosomes (48). A majority of the sites with increased NF-Y accessibility occurred in LXR-binding genes. More directed studies are needed to explore the mechanistic relationships between LXR and NF-Y.

Prior studies have documented instances of squelching, in which an activated transcription factor represses a target gene without binding to its location by competing for cofactors (49–52). However, our study was not designed to test this mode of regulation for LXRs, as we did not perform ATAC-seq in the presence of synthetic LXR agonist treatment. For genes up-regulated in LXRDKO liver that have no direct LXR binding, we observed an enrichment of the CTCF motif in peaks that lost accessibility and NF-Y motif in peaks that gained accessibility. This finding suggests that changes in CTCF and NF-Y may contribute to the ability of LXR to repress genes without direct binding. The mechanism whereby loss of LXR alters CTCF and NF-Y activity on LXR-binding and -nonbinding genes requires further investigation.

## Methods

### Mice.

*Lxr*α^−/−^ and *Lxr*β^−/−^ mice originally provided by David Mangelsdorf (University of Texas Southwestern Medical Center, Dallas, TX) were backcrossed more than 10 generation to the C57/Bl6 background. Animals were housed in a 25 °C temperature-controlled room under a 12-h light/12-h dark cycle under pathogen-free conditions. Mice had ad libitum access to water and standard chow (Harlan NIH-31, 3.1 kcal/g, 23% calories from protein, 18% from fat, and 59% from carbohydrate). Mice were killed at 8 wk of age. All animal experiments were approved by the Institutional Animal Care and Research Advisory Committee of the University of California, Los Angeles.

### RNA-Seq Sample Preparation.

RNA from frozen tissue was extracted through TRIzol (Invitrogen) and a Qiagen RNeasy Mini Kit. Total RNA libraries were made with a KAPA Stranded kit with mRNA capture. Libraries were sequenced as single end (50 bp) on an Illumina HiSeq3000.

### RNA-Seq Data Processing and Analysis.

Data quality analysis was performed via FastQC (53). The reads were aligned to the mm10 genome using STAR (version 2.6.0c) (54). Alignments were visualized using samtools (55) and the IGV browser (56). Differential expression analysis was performed with DESeq2 (57), and genes were classified as significantly regulated if adjusted *P* value <0.05. Genes were annotated using *biomaRt* package in R (https://www.R-project.org/) (58). Plots and heatmaps were created in R using *pheatmap* and *EnhancedVolcano* and the *ClustVis* web tool (59, 60). Gene sets were enriched for pathways using *BioPlanet* 2019 and ChIP-seq targets using ChIP enrichment analysis (ChEA) through *Enrichr* (61–63).

### ATAC-Seq Sample Preparation.

ATAC-Seq from tissue was conducted as previously published (64) with some modifications. Approximately 50 to 100 mg of fresh tissue was homogenized via a dounce homogenizer in 1 mL of nuclear isolation buffer (20 mM Tris⋅HCl, 50 mM ethylenediaminetetraacetic acid, 5 mM spermidine, 0.15 mM spermine, 0.1% mercaptoethanol, 40% glycerol, 1 mM egtazic acid, 60 mM KCl, 1% octylphenoxypolyethoxyethanol, pH 7.5) and filtered through a 40-μM nylon filter. Samples were centrifuged at 4 °C at 1,000 × *g* for 10 min and the pellet was resuspended with 1 mL cold resuspension buffer (RSB) (10 mM Tris⋅HCl, 10 mM NaCl, 3 mM MgCl2, pH 7.4). Approximately 50,000 nuclei from these samples were removed and centrifuged at 4 °C at 500 × *g* for 5 min. Supernatant was removed and the transposase reaction was performed immediately as described (65). DNA was purified using a Qiagen MinElute Kit and libraries were prepared as described (65). Size selection was done with AMPure XP magnetic beads. Libraries were quantified by qPCR using NEBNext Library Quant Kit for Illumina and sequenced on Illumina HiSEq 4000 as single-end 50 bp at the University of California, Los Angeles (UCLA) Broad Stem Cell Research Center Sequencing core.

### ATAC-Seq Data Processing and Analysis.

Samples were demultiplexed and quality control was done using FastQC (53). Cutadapt (66) was used to trim adapters and trimmed sequences were aligned to the mm10 mouse genome assembly using bowtie2 (67). Mitochondrial, unmapped, multimapped, and duplicate reads were removed using *samtools* (55) and in-house scripts. Peaks were called using MACS2 (68) and quantitated across samples using Seqmonk (69) generating reads per kilobase per million mapped reads (RPKM). Peaks were annotated to genomic features and nearest promoter via the Homer (70) *annotate* function. *Bedgraphs* were created using Homer and converted to .tdf files for visualization in the IGV browser (56). tSNE plots were created using Seqmonk. For ranked analysis, peaks that had fewer than 10 counts across all four samples were removed. We ran the Irreproducible Discovery Rate (IDR) software for quality control (37). The filtering improved the percentage of peaks that met the 1e-6 threshold in the IDR software. The reads from replicates for each peak were averaged and the peaks were ranked based on the difference between the average counts among conditions. The *pheatmap* R package was used to plot the top 1,000 peak heatmap. ChIP-Seeker was used to plot the distribution of peaks relative to TSSs (71). Merged bam files were created using the *samtools* merge function. deepTools2 was employed to profile the signal intensity across defined peaks using the merged replicates (72).

Motif analysis to infer TF binding was done through the *findMotifGenome* and *findMotifs* functions in Homer using known motifs. Ranked peaks were binned into equal-sized bins and known motif analysis was run for each bin. The *P* value for each motif was plotted across all bins. Nonenriched and not-changing motifs were filtered out. Motifs with high similarity (>0.90) within the same TF family were combined. Footprinting was done with Hmm-based identification of transcription factor footprints (HINT)-ATAC using the bam files and the JASPAR motif database as input (38, 73).

### Additional ChIP-Seq and RNA-Seq Datasets.

Additional data were downloaded from the Gene Expression Omnibus and processed as above: LXR liver ChIP-Seq data (GSE35262), LXR vehicle ChIP-Seq data (GSM864670), and LXR T09 peaks (GSM864669). Differentially expressed genes in response to LXR agonist treatment were obtained from GSE149075. Hepatic SREBP-2 peaks were obtained from GSE28082. The ChIP-Atlas was used to provide a summarized ChIP-Seq experiment from mouse liver or hepatocytes or liver-derived cell lines (74).

### Validation with LXR Agonist.

Nine-week-old mice on mixed background 129 × 1/SvJ and C57BL/6 were gavaged with 40 mg/kg GW3965 (75) first 17 h before and second 8 h before killing. Mice were 4 h fasted before killing. GW3965 was gavaged in canola oil. Dimethylsulfoxide was used as vehicle control. RNA was extracted using TRIzol. The differences between gene expression were determined via qPCR using Taq Universal SYBR Green Supermix (Bio-Rad) using primers that are provided in *SI Appendix*, Table S1.

## Supplementary Material

Supplementary File

## Data Availability

ATAC-Seq and RNA-Seq data have been deposited to the Gene Expression Omnibus (GEO) database (GSE191030) (76).
