# Supplementary material for: Integrative analysis reveals multiple modes of LXR transcriptional regulation in liver
Source: Proc Natl Acad Sci U S A. 2022 Feb 10;119(7):e2122683119. doi: 10.1073/pnas.2122683119 (PMC8851562; doi:10.1073/pnas.2122683119)
Supplement: Supplementary File [file pnas.2122683119.sapp.pdf]

Supplemental Figure Legends

SI Appendix Figure S1: Transcriptional changes in LXR-deficient liver.

A. Differential expression analysis of the LXRDKO liver RNA-Seq results showed 246 upregulated and 321 downregulated genes. B. Volcano plot showing differential gene expression between WT and LXRDKO samples. Genes are color-coded based on their fold change and adjusted p value. C. Top 10 downregulated (left) and upregulated (right) genes from LXRDKO liver RNA-Seq based on adjusted p value.

SI Appendix Figure S2: Expression profiles of LXRDKO livers.

A. Pathway enrichment results for downregulated (top) and upregulated (bottom) genes in LXRDKO liver. B. Transcription Factor ChIP-Seq enrichment analysis (ChEA) for downregulated genes in LXRDKO liver. Publicly available ChIP-Seq data were used to estimate enrichment of transcription factor binding in the promoters of genes downregulated in LXRDKO liver.

SI Appendix Figure S3: Chromatin accessibility profiles of LXRDKO livers

A. Correlation heatmap of ATAC-Seq samples using all peaks. B. t-SNE plot of ATAC-Seq samples. C. Distribution of top 1000 peaks with largest loss and gain in LXRDKO liver based on relative distance to closest TSS. D. Average change in accessibility of peaks associated with differentially expressed genes in LXRDKO liver. E. Average change in accessibility in intronic (left), intergenic (right), and promoter (bottom) regions for all genes (All) and those whose expression was reduced (Down) or increased (Up) in LXRDKO livers. * comparison to all genes; # comparison between downregulated and upregulated genes.

SI Appendix Figure S4: Pathway enrichment.

Pathway enrichment analysis of the top 1000 peaks that lost (top) and gained (bottom) accessibility.

SI Appendix Figure S5: Changes to LXR binding sites in LXRDKO livers.

A. Proportion of LXR basal or agonist induced binding genes among genes differentially expressed in LXRDKO livers. B. Average number of (basal) LXR binding sites per gene for all LXR target genes and genes that are differentially expressed in LXRDKO liver. C. Changes in accessibility across LXR binding sites in LXRDKO liver compared to WT. D. Proportion of LXR binding sites within intergenic, intronic and promoter regions plotted as a function of the change in accessibility in LXRDKO liver.

SI Appendix Figure S6: Loss of LXRs reduces motif accessibility of other transcription factors.

A. Heatmaps of motif enrichment of selected transcription factors from Figure 4A across binned intergenic, intronic and promoter ATAC-Seq peaks based on the change in accessibility. B. ATAC-Seq signal intensity heatmap and profile across peaks with PPARE motif, among the top 1000 peaks losing accessibility in LXRDKO livers. C. (Left) Hierarchical clustering heatmap for

transcription factor motif co-occurrence among the least accessible 10 bins from Figure 4A. Eachcolumn represents a peak and red indicates presence of the corresponding motif in that peak. (Right) Hierarchical clustering heatmap for transcription factor motif co-occurrence among LXR binding genes that are associated with the least accessible 10 bins in LXRDKO livers. Each column represents a gene and red indicates presence of the corresponding motif in the peaks associated with the same gene. D. Normalized counts for the expression of transcription factors identified in Figure 4A. (* is used to indicate the FDR < 0.05)

SI Appendix Figure S7: Impact of the loss of LXR on other transcription factor motif activity.

Footprint profile of HNF6 (A) and HNF1B (B) using the ATAC-Seq samples for LXRDKO and WT with merged replicates. C. Pathway enrichment analysis of genes associated with increased accessibility of the NFY motif (top 10 bin from Figure 5A). D. Volcano plot of the expression profile of genes with NFY motif containing peaks within the top 10 bins that gain accessibility (from Figure 5A).

SI Appendix Figure S8: Change in accessibility and genomic features at the LXR binding sites in relation to different modes of LXR action.

A. Proportions of genomic annotations for LXR binding peaks that are associated with genes that are differentially upregulated or downregulated in LXRDKO and either downregulated, upregulated or not changing in response to either GW3965 and T0901317 treatment are plotted. B. Pathway enrichment analysis of genes that were downregulated in LXRDKO and have an LXR ChIP-Seq peak but did not change in expression in response to agonist treatment. C. LXR ChIP-Seq (basal) sites are segregated based on their genomic feature and ranked and binned based on their change in accessibility in LXRDKO livers. Motif analysis was performed for each of the bins across the accessibility and genomic features. Similar motifs were combined (>.90 Similarity) and motif with low enrichment were not displayed. Transcription factor motifs enriched in the LXR binding intergenic (left), intron (middle) and promoter (right) regions are displayed. D. Signal intensity heatmap and profiles of ATAC peaks at LXR binding sites proximal to ligand-dependent repressed genes.

A

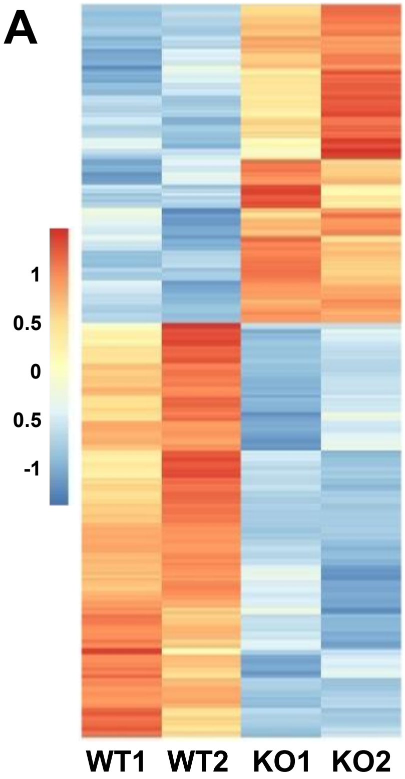

B

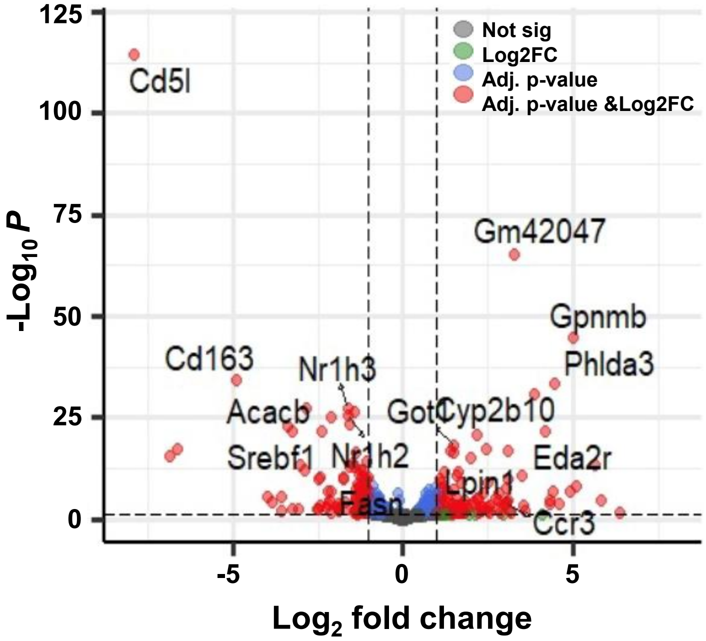

C

Top 10 Downregulated Genes

Name	Adj. p value	Fold Change	Type
Cd5l	2.05E-115	0.0040	protein coding
Cd163	4.39E-35	0.0335	protein coding
Clec4f	3.45E-28	0.1402	protein coding
Nr1h3	3.45E-28	0.3239	protein coding
Ces1e	2.66E-27	0.3733	protein coding
Nr1h2	3.87E-26	0.3219	protein coding
Acacb	5.26E-26	0.2306	protein coding
1810008I18Rik	3.91E-24	0.3331	lincRNA
Hamp2	9.02E-24	0.0939	protein coding
Srebf1	1.84E-22	0.1875	protein coding

Top 10 Upregulated Genes

Name	Adj. p value	Fold Change	Type
Gm42047	4.36E-66	9.6148	lincRNA
Gpnmb	1.44E-45	32.1408	protein coding
Phlda3	3.49E-34	21.7534	protein coding
Sult2a7	1.15E-31	14.4144	protein coding
Eda2r	2.66E-22	18.0955	protein coding
Cyp2b10	2.70E-21	4.4866	protein coding
Got1	1.20E-18	2.8311	protein coding
Cplane1	4.55E-18	5.3950	protein coding
Gm48199	9.53E-18	8.6393	lincRNA
Fkbp5	9.54E-18	2.6513	protein coding

Figure S1

A

Downregulated Pathwaysz	Adjusted p-value	Genes
PPAR signaling pathway	3.92E-08	*Acsl5, Lpl, Nr1h3, Dbi, Sorbs1, Fabp1, Fads2, Fabp2, Fabp5, Acox1, Me1, Cd36, Ppara*
Lipid and lipoprotein metabolism	2.11E-06	*Slc44a1, Mttp, Lpl, Sgms2, Acacb, Acaca, Mtm1, Helz2, Fads2, Me1, Cd36, Arsa, Srebf1, Stard4, Hsd3b2, Elovl5, Srd5a1, Slc10a2, Elovl2, Acsl5, Fabp1, Gpam, Acox1, Fasn, Lpcat3, Ppara, Mgll, Abcg1*
Fatty acid, triacylglycerol, and ketone body metabolism	1.70E-04	*Srebf1, Elovl5, Elovl2, Acsl5, Acacb, Acaca, Helz2, Fabp1, Gpam, Acox1, Fasn, Me1, Cd36, Ppara*

Upregulated Pathways	Adjusted P-value	Genes
Cysteine and methionine metabolism	0.001861	*Sds, Got1, Tat, Cth, Mat1a, Cdo1*
p53 activity regulation	0.004402	*Cdkn1a, Zmat3, Igfbp3, Sesn2, Ccng1, Mdm2, Tnfrsf10b, Bax, Gtse1*
MicroRNA regulation of DNA damage response	0.004582	*Cdkn1a, Myc, Ccng1, Mdm2, Tnfrsf10b, Bax, Brca1*

B

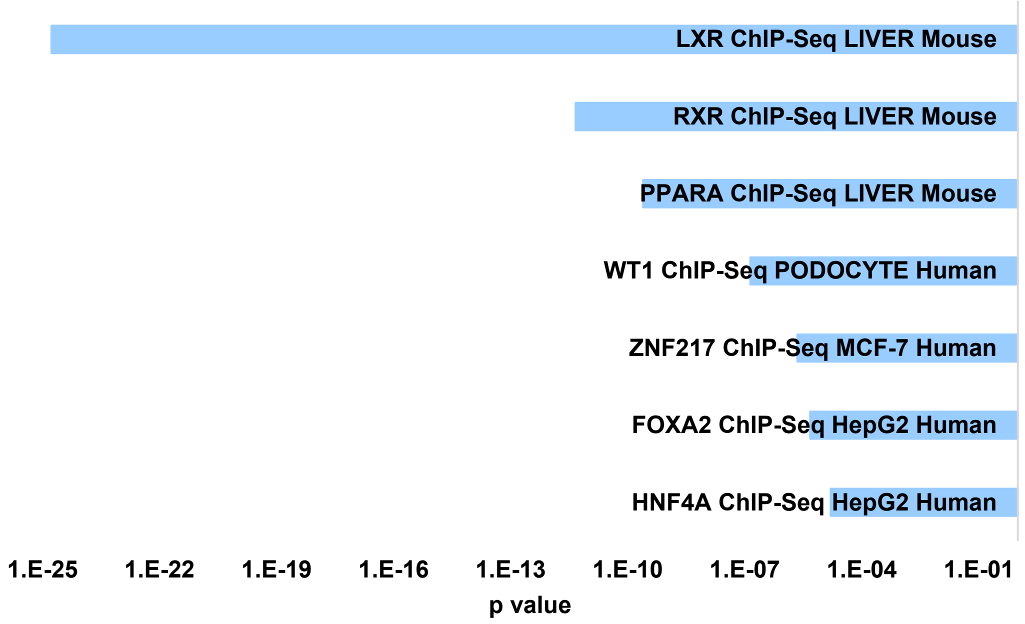

Figure S2

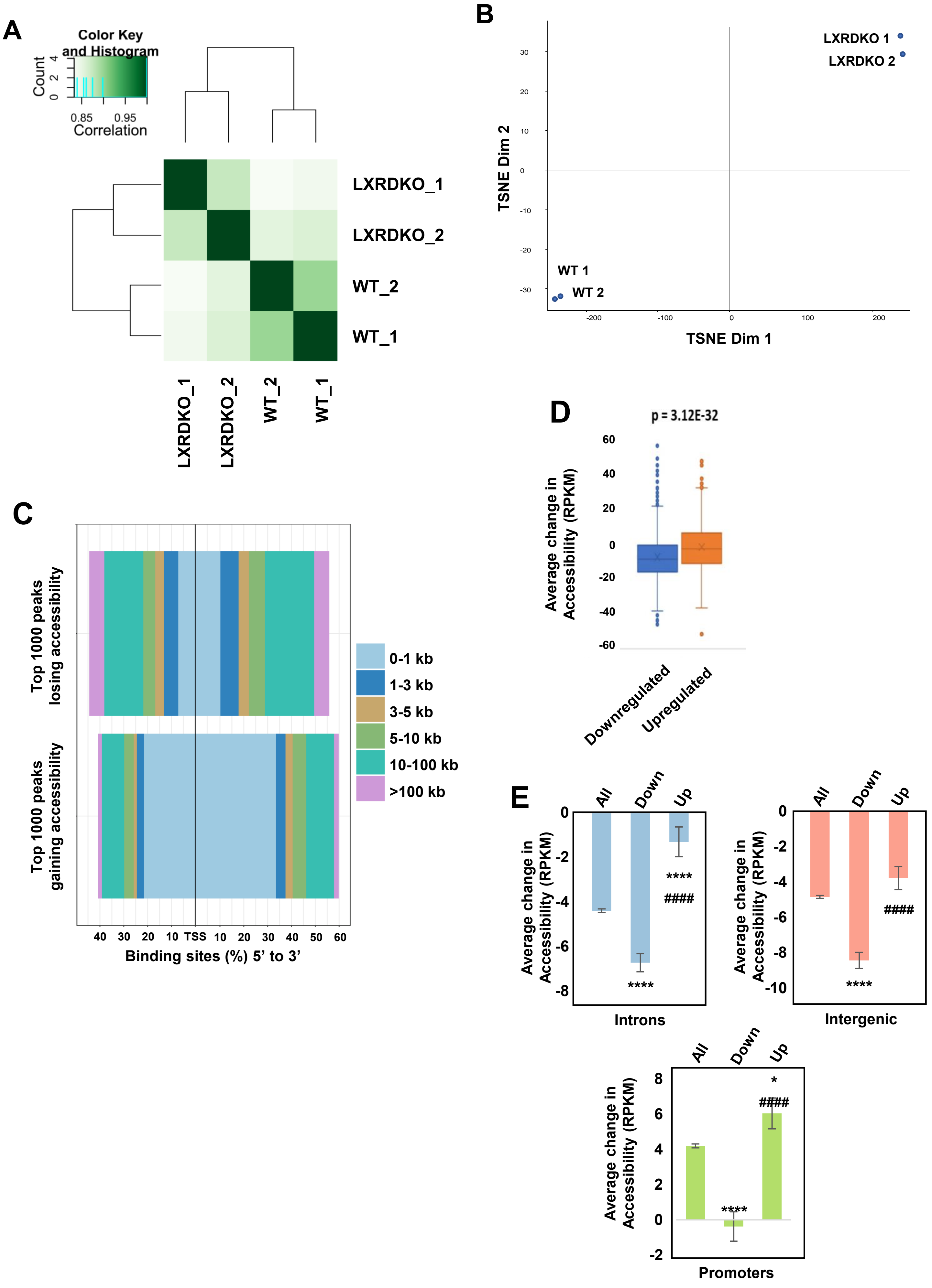
A
Color Key and Histogram
Count
0.85
0.95
Correlation
LXRDKO_1
LXRDKO_2
WT_2
WT_1
B
LXRDKO 1
LXRDKO 2
WT 1
WT 2
TSNE Dim 2
TSNE Dim 1
C
Top 1000 peaks losing accessibility
Top 1000 peaks gaining accessibility
0-1 kb
1-3 kb
3-5 kb
5-10 kb
10-100 kb
>100 kb
Binding sites (%) 5' to 3'
D
p = 3.12E-32
Average change in Accessibility (RPKM)
Downregulated
Upregulated
E
All
Down
Up
Introns
Intergenic
Promoters
****
####
*

Figure S3

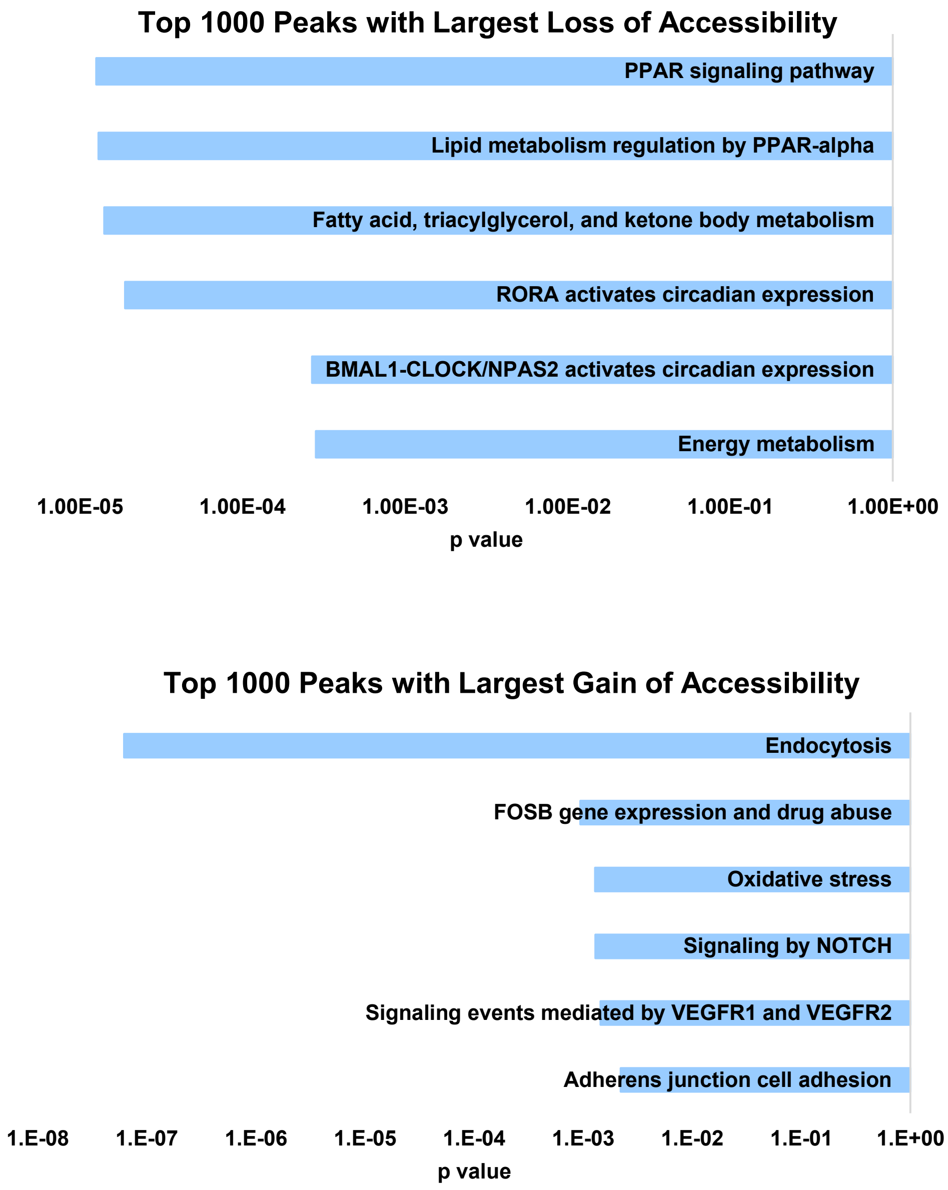
Top 1000 Peaks with Largest Loss of Accessibility
PPAR signaling pathway
Lipid metabolism regulation by PPAR-alpha
Fatty acid, triacylglycerol, and ketone body metabolism
RORA activates circadian expression
BMAL1-CLOCK/NPAS2 activates circadian expression
Energy metabolism
1.00E-05
1.00E-04
1.00E-03
1.00E-02
1.00E-01
1.00E+00
p value
Top 1000 Peaks with Largest Gain of Accessibility
Endocytosis
FOSB gene expression and drug abuse
Oxidative stress
Signaling by NOTCH
Signaling events mediated by VEGFR1 and VEGFR2
Adherens junction cell adhesion
1.E-08
1.E-07
1.E-06
1.E-05
1.E-04
1.E-03
1.E-02
1.E-01
1.E+00
p value

Figure S4

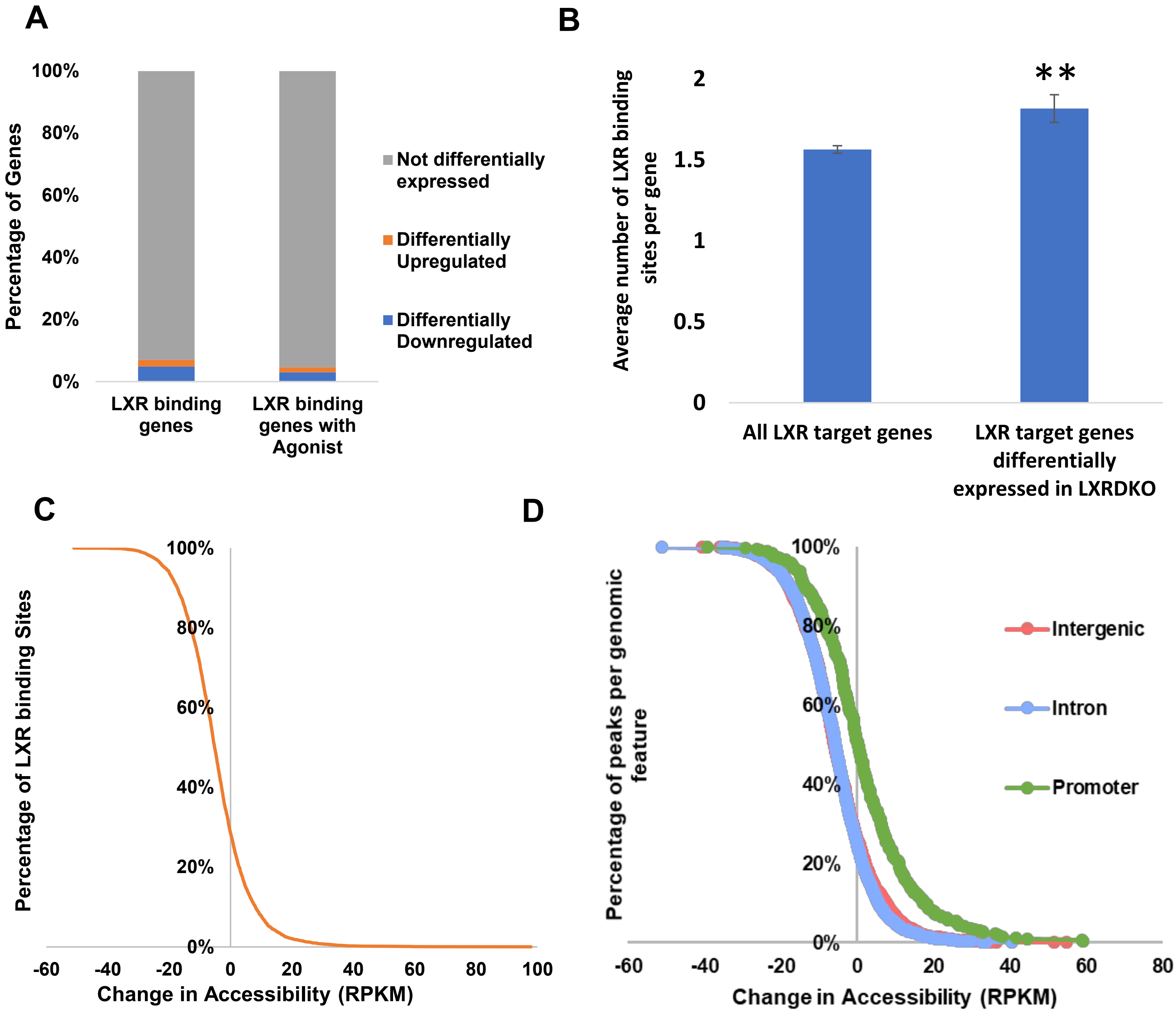
A
100%
80%
60%
40%
20%
0%
Percentage of Genes
LXR binding genes
LXR binding genes with Agonist
Not differentially expressed
Differentially Upregulated
Differentially Downregulated
B
**
2
1.5
1
0.5
0
Average number of LXR binding sites per gene
All LXR target genes
LXR target genes differentially expressed in LXRDKO
C
Percentage of LXR binding Sites
100%
80%
60%
40%
20%
0%
-60
-40
-20
0
20
40
60
80
100
Change in Accessibility (RPKM)
D
Percentage of peaks per genomic feature
100%
80%
60%
40%
20%
0%
-60
-40
-20
0
20
40
60
80
Change in Accessibility (RPKM)
Intergenic
Intron
Promoter

Figure S5

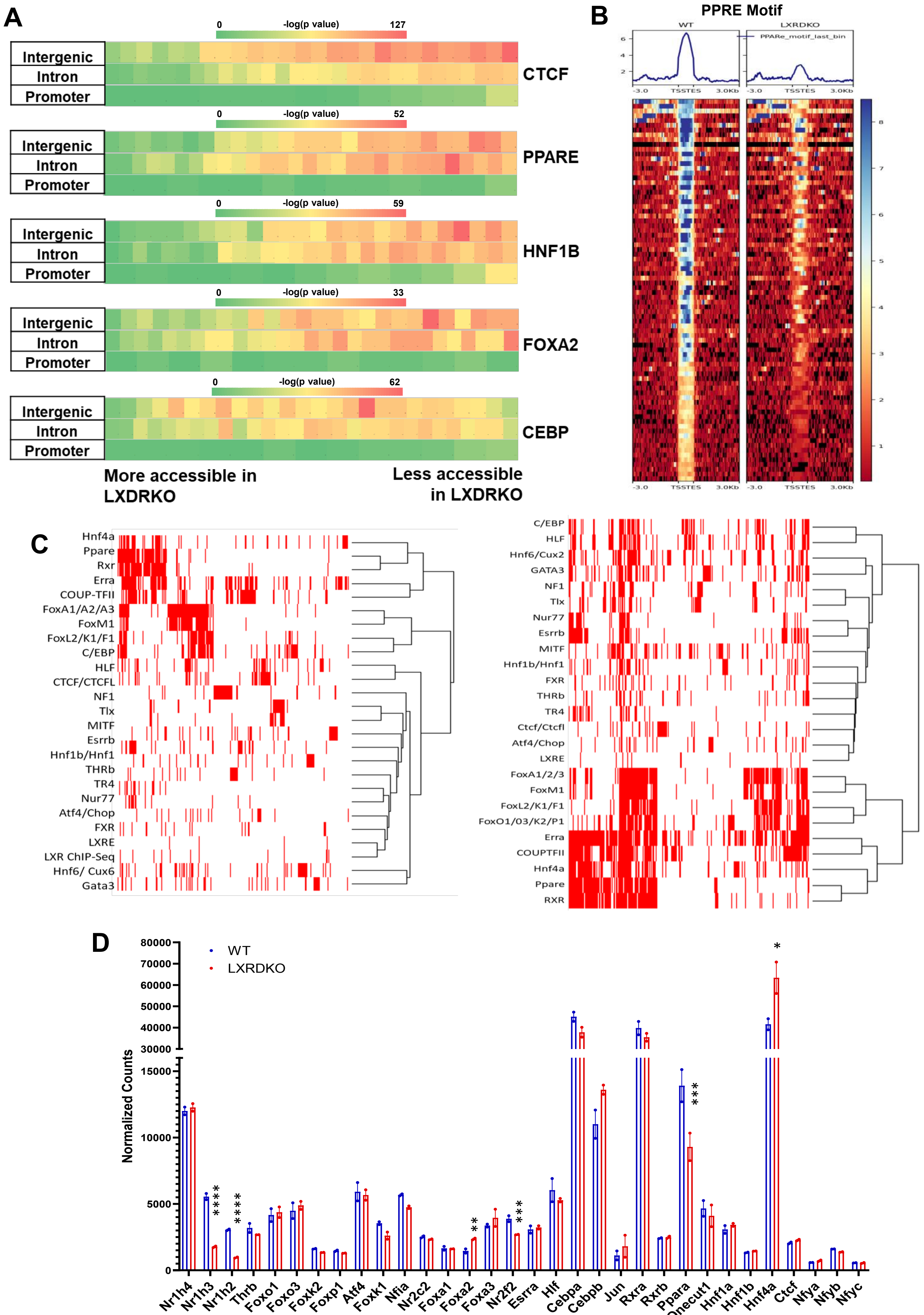
A
B
C
D
-log(p value)
Intergenic
Intron
Promoter
CTCF
PPARE
HNF1B
FOXA2
CEBP
More accessible in LXDRKO
Less accessible in LXDRKO
PPRE Motif
WT
LXRDKO
Normalized Counts
WT
LXRDKO
Nr1h4
Nr1h3
Nr1h2
Thrb
Foxo1
Foxo3
Foxk2
Foxp1
Atf4
Foxk1
Nfia
Nr2c2
Foxa1
Foxa2
Foxa3
Nr2f2
Esrra
Hlf
Cebpa
Cebpb
Jun
Rxra
Rxrb
Ppara
Onecut1
Hnf1a
Hnf1b
Hnf4a
Ctcf
Nfya
Nfyb
Nfyc

Figure S6

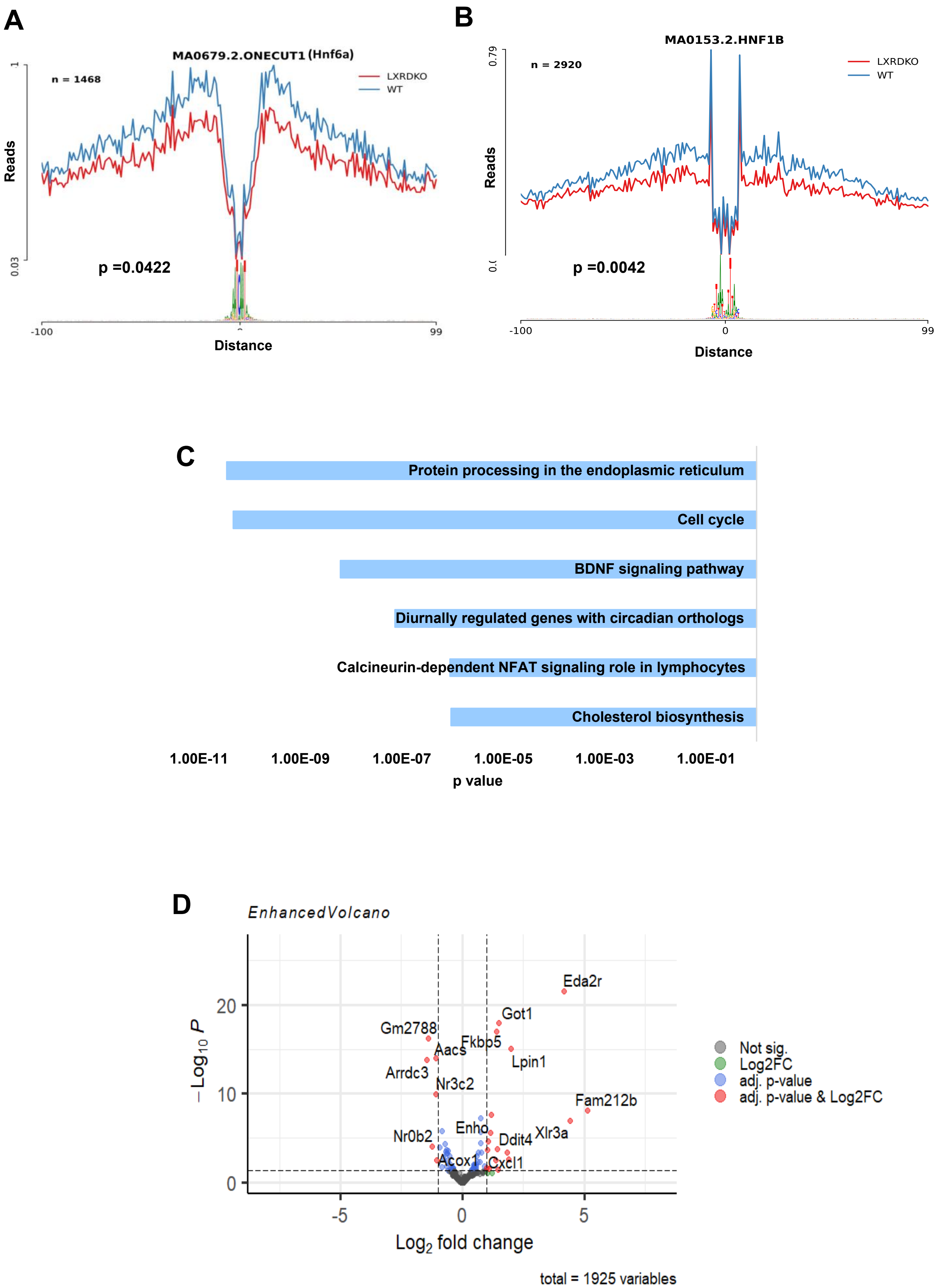
A
MA0679.2.ONECUT1 (Hnf6a)
n = 1468
LXRDKO
WT
Reads
p =0.0422
-100
99
Distance
B
MA0153.2.HNF1B
n = 2920
LXRDKO
WT
0.79
Reads
p =0.0042
-100
0
99
Distance
C
Protein processing in the endoplasmic reticulum
Cell cycle
BDNF signaling pathway
Diurnally regulated genes with circadian orthologs
Calcineurin-dependent NFAT signaling role in lymphocytes
Cholesterol biosynthesis
1.00E-11
1.00E-09
1.00E-07
1.00E-05
1.00E-03
1.00E-01
p value
D
EnhancedVolcano
Eda2r
Got1
Gm2788
Fkbp5
Aacs
Lpin1
Arrdc3
Nr3c2
Fam212b
Enho
Nr0b2
Ddit4
Xlr3a
Acox1
Cxcl1
20
10
0
-5
0
5
$-\mathrm{Log}_{10}\,P$
Log_2 fold change
Not sig.
Log2FC
adj. p-value
adj. p-value & Log2FC
total = 1925 variables

Figure S7

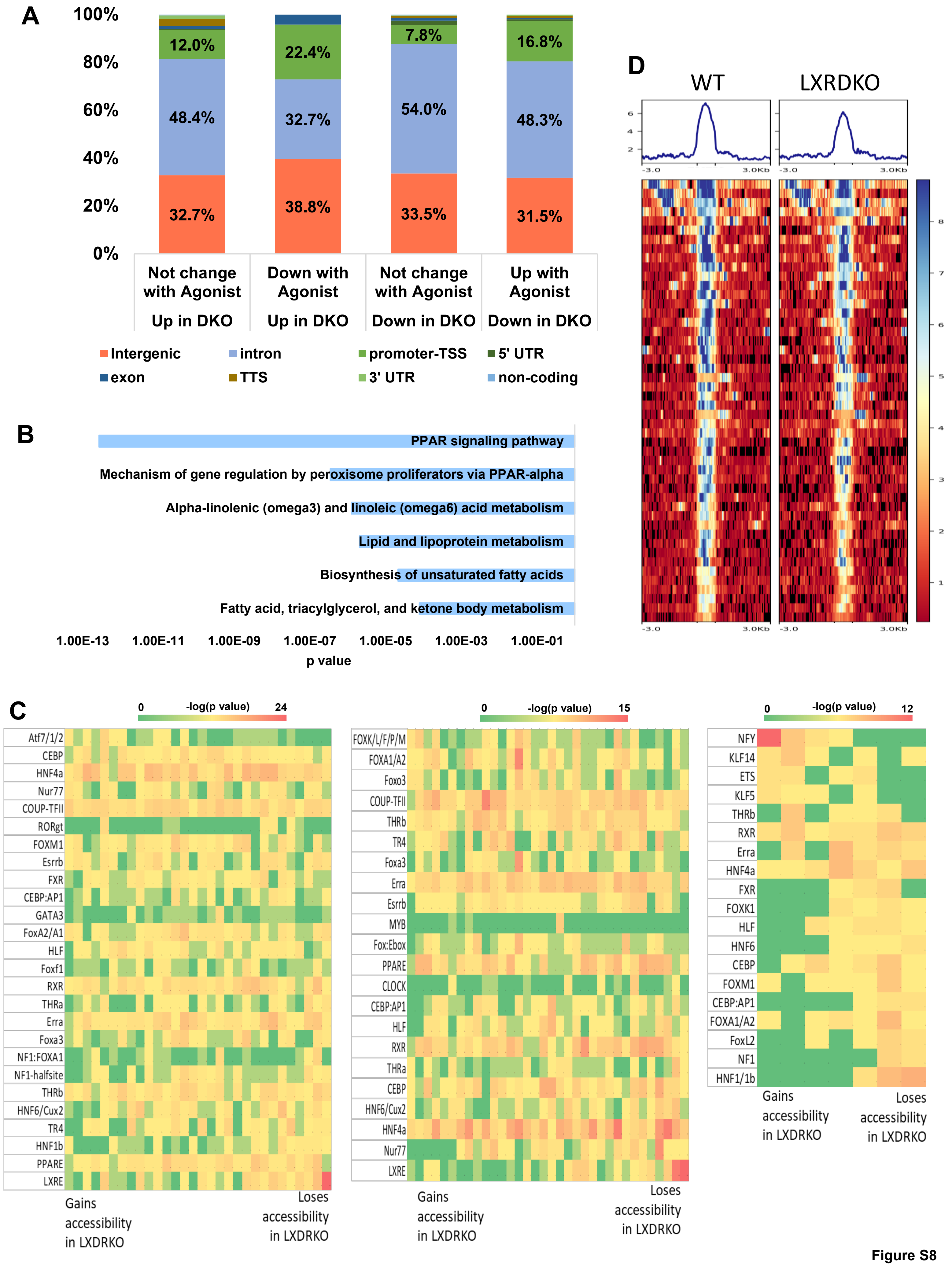
A
100%
80%
60%
40%
20%
0%
12.0%
48.4%
32.7%
22.4%
32.7%
38.8%
7.8%
54.0%
33.5%
16.8%
48.3%
31.5%
Not change with Agonist
Up in DKO
Down with Agonist
Up in DKO
Not change with Agonist
Down in DKO
Up with Agonist
Down in DKO
Intergenic
intron
promoter-TSS
5' UTR
exon
TTS
3' UTR
non-coding
B
PPAR signaling pathway
Mechanism of gene regulation by peroxisome proliferators via PPAR-alpha
Alpha-linolenic (omega3) and linoleic (omega6) acid metabolism
Lipid and lipoprotein metabolism
Biosynthesis of unsaturated fatty acids
Fatty acid, triacylglycerol, and ketone body metabolism
1.00E-13
1.00E-11
1.00E-09
1.00E-07
1.00E-05
1.00E-03
1.00E-01
p value
D
WT
LXRDKO
-3.0
3.0Kb
C
0
-log(p value)
24
Atf7/1/2
CEBP
HNF4a
Nur77
COUP-TFII
RORgt
FOXM1
Esrrb
FXR
CEBP:AP1
GATA3
FoxA2/A1
HLF
Foxf1
RXR
THRa
Erra
Foxa3
NF1:FOXA1
NF1-halfsite
THRb
HNF6/Cux2
TR4
HNF1b
PPARE
LXRE
Gains accessibility in LXDRKO
Loses accessibility in LXDRKO
0
-log(p value)
15
FOXK/L/F/P/M
FOXA1/A2
Foxo3
COUP-TFII
THRb
TR4
Foxa3
Erra
Esrrb
MYB
Fox:Ebox
PPARE
CLOCK
CEBP:AP1
HLF
RXR
THRa
CEBP
HNF6/Cux2
HNF4a
Nur77
LXRE
Gains accessibility in LXDRKO
Loses accessibility in LXDRKO
0
-log(p value)
12
NFY
KLF14
ETS
KLF5
THRb
RXR
Erra
HNF4a
FXR
FOXK1
HLF
HNF6
CEBP
FOXM1
CEBP:AP1
FOXA1/A2
FoxL2
NF1
HNF1/1b
Gains accessibility in LXDRKO
Loses accessibility in LXDRKO

Figure S8

Supplemental Table 1. PCR Primers used

Primer name (m f	Sequence
mLpin1_F	CATGCTTCGGAAAGTCCTTCA
mLpin1_R	GGTTATTCTTTGGCGTCAACCT
mCxcl1_F	CTGGGATTCACCTCAAGAACATC
mCxcl1_R	CAGGGTCAAGGCAAGCCTC
mSaa4_F	CTCTGTTCTTTGTTCCTGGGAG
mSaa4_R	CTAGGTTGTCCCGATAGGCTC
mSlc25a15_F	GCTGCCTCAAGACCTACTCC
mSlc25a15_R	CCGTAACACATGAACAGCACC
mSorbs3_F	TTCAGCTTCGTCTTTGAACAACA
mSorbs3_R	CTTGGGTCAAGGTTGGAGGA
mNnmt_F	TGTGCAGAAAACGAGATCCTC
mNnmt_R	AGTTCTCCTTTTACAGCACCCA
mCdkn1a_F	CCTGGTGATGTCCGACCTG
mCdkn1a_R	CCATGAGCGCATCGCAATC
mTbc1d8_F	AGCCTAGCCAGATCACAAAGA
mTbc1d8_R	CGTCCAGAGGGAACAGTCT
mTymp_F	CGCGGTGATAGATGGAAGAGC
mTymp_R	CACACCTCCTGTGGAGTGTT
mGot1_F	GCGCCTCCATCAGTCTTTG
mGot1_R	ATTCATCTGTGCGGTACGCTC
mEtnppl_F	GCTCTCCGTTTGCTACTTCAC
mEtnppl_R	CCCTCTTGACATCTTTGCCCTT
mIl1r1_F	CGAACCGTGAACAACACAAA
mIl1r1_R	CAGAGGCACCATGAGACAAA
mLurap1l_F	TCTCTTGGGTCTCTCGGTATAA
mLurap1l_R	TCCACAGCCAGCAAGATTAG
mRetreg1_F	GCCATCAAAGACCAGCTAGAA
mRetreg1_R	GTCCCAGCTCACTCTCAATTT
